# RETRACTION: Impact of Robotic and Open Surgery on Patient Wound Complications in Gastric Cancer Surgery: A Meta‐Analysis

**DOI:** 10.1111/iwj.70338

**Published:** 2025-03-18

## Abstract

**RETRACTION:** YeL. , YangQ. , XueY. , JiaR. , YangL. , ZhongL. , ZouL. , and XieY. , “Impact of Robotic and Open Surgery on Patient Wound Complications in Gastric Cancer Surgery: A Meta‐Analysis,” International Wound Journal 20, no. 10 (2023): 4262–4271, 10.1111/iwj.14328.37496310 PMC10681412

The above article, published online on 26 July 2023, in Wiley Online Library (http://onlinelibrary.wiley.com/), has been retracted by agreement between the journal Editor in Chief, Professor Keith Harding; and John Wiley & Sons Ltd. Following an investigation by the publisher, all parties have concluded that this article was accepted solely on the basis of a compromised peer review process. The editors have therefore decided to retract the article. The authors did not indicate their agreement with the retraction.

**RETRACTION:** L. Ye , Q. Yang , Y. Xue , R. Jia , L. Yang , L. Zhong , L. Zou , and Y. Xie , “Impact of Robotic and Open Surgery on Patient Wound Complications in Gastric Cancer Surgery: A Meta‐Analysis,” International Wound Journal 20, no. 10 (2023): 4262–4271, 10.1111/iwj.14328.37496310 PMC10681412

The above article, published online on 26 July 2023, in Wiley Online Library (http://onlinelibrary.wiley.com/), has been retracted by agreement between the journal Editor in Chief, Professor Keith Harding; and John Wiley & Sons Ltd. Following an investigation by the publisher, all parties have concluded that this article was accepted solely on the basis of a compromised peer review process. The editors have therefore decided to retract the article. The authors did not indicate their agreement with the retraction.

